# Cultural adaptation and psychometric properties of the online learning climate scale for Chilean university students

**DOI:** 10.3389/fpsyg.2024.1280311

**Published:** 2024-02-14

**Authors:** Mónica Bravo-Sanzana, Rafael Miranda, Oscar Terán-Mendoza, Manuel Mieres-Chacaltana, Luis Carabantes

**Affiliations:** ^1^Núcleo Científico y Tecnológico en Ciencias Sociales y Humanidades, Universidad de La Frontera, Temuco, Chile; ^2^Departamento de Psicología, Universidad Continental, Huancayo, Peru; ^3^Doctorado en Psicología, Universidad de La Frontera, Temuco, Chile; ^4^Departamento de Diversidad y Educación Intercultural, Universidad Católica de Temuco, Temuco, Chile; ^5^School of Languages, Linguistics and Film, Queen Mary University of London, London, United Kingdom

**Keywords:** online learning, classroom climate, distance education, psychometric analysis, reliability, validity

## Abstract

**Introduction:**

The COVID-19 pandemic has profoundly changed university teaching and learning formats, leading to a significant increase in online learning. Consequently, the crisis has facilitated the potential development of this educational modality. However, researchers need adapted and validated instruments to assess the online learning climate in universities.

**Aim:**

This study aimed to adapt and psychometrically validate the Online Learning Climate Scale (OLCS) for Chilean university students.

**Method:**

Quantitative research was conducted with a non-experimental and cross-sectional, design executed in two phases: the first was oriented to the cultural adaptation of the instrument, and the second was focused on analyzing its psychometric properties in a sample of 491 university students.

**Results:**

A translated and culturally adapted version was obtained, composed of 15 items distributed in a factorial structure composed of four dimensions that showed excellent adjustment to the data [*χ*^2^ (84) = 189.628; *p* < 0.001; CFI = 0.979; TLI = 0.973; RMSEA = 0.051 (IC90% 0.044–0.059); SRMR = 0.028]; internal consistency was estimated through Cronbach’s alpha and ranged between 0.892 and 0.955, and strict invariance between men and women was achieved.

**Discussion:**

The Online Learning Climate Scale (OLCS) is a valid and reliable measure for measuring the online learning climate within the Chilean higher education context so that it can be used both in research and in monitoring management programs in educational environments.

## Introduction

The pandemic associated with the SARS-CoV-2 virus drastically transformed various areas of people’s lives worldwide, including educational environments (Aristovnik et al., 2020). To reduce the spread of the virus, universities worldwide had to rapidly transform face-to-face courses to virtual mode (Ali, 2020; Sahu, 2020). However, for many students, virtual teaching is strange and even threatening because it requires higher levels of self-regulation to achieve optimal learning outcomes (Aristovnik et al., 2020), especially when considering social interaction through participation in online discussions or group work (Thomas, 2012; Whittaker, 2015).

Some students adapt more quickly than others to virtual learning environments and promptly handle the ambiguities and uncertainties that these scenarios may represent (Cole et al., 2019). Adaptability to a virtual classroom environment depends on several factors. Still, one of the main ones is the learning climate, which is conceptualized as the bonding, synchronization, and interaction that is generated between students, instructors, and the course structure from the social relationships that occur within the context of synchronous or asynchronous learning on digital platforms (Derakhshesh et al., 2022). Considering that virtual learning environments entail different challenges than traditional face-to-face teaching (e.g., geographical separation between participants), it has been of interest to other research groups to determine the components of the construct and to analyze its relationship with outcomes of interest within institutions such as well-being and academic achievement (Johnson, 2006; Wang et al., 2020).

So far, most of the research in this area has been conducted primarily in the United States, Europe, and Australia, with few reports of research conducted in other parts of the world (Stanley and Montero Fortunato, 2022). This is particularly relevant when considering that online learning does not have the same meaning in different cultures, given that cultural values influence elements of instruction, collaboration, and academic behavior (Liu et al., 2010). Therefore, a vital issue is to have culturally sensitive and psychometrically validated instruments to measure and assess the online learning climate in higher education students, especially in broader contexts, such as Latin American countries, where research is very recent and challenging (Okoye et al., 2023). This article will propose and discuss the theoretical and empirical background highlighting the need to adapt the Online Learning Climate Scale (OLCS) linguistically and culturally to assess the online teaching climate in higher education.

### Online classroom climate

The concept of classroom climate in face-to-face settings (hereafter, “traditional classroom climate”) is understood as the perceived connection between instructor and students (Cooper, 1995; Myers, 1995; Dwyer et al., 2004). However, Frisby and Martin (2010) describe classroom climate as the perception of connectedness and includes course organization, arguing that the perceived relationship with instructors and classmates is related to perceptions of connectedness in the classroom. López González and Bisquerra Alina (2013) have indicated that classroom climate involves organization, performance, and socio-affective quality.

It is worth noting that while there is evidence of traditional classroom climate, less is known about what constitutes the learning climate in online environments for university students. Online learning combines synchronous and asynchronous classes where instructors and students are physically separated (Brophy et al., 2021). Synchronous courses are delivered using computer-based tools, while asynchronous classes correspond to self-study that can be supplemented with interactions via email and platforms (Bogolepova, 2021). Unlike face-to-face courses, Kaufmann et al. (2015) have posited that interaction between instructors and students in online courses can occur asynchronously, synchronously, or in a mixed form. However, at an early stage, educators have raised concerns about the lack of communication and participation in the online learning environment (Allen, 2006).

### Theoretical approach to online learning

The instructional beliefs model (IBM) provides a conceptual framework that enables a clear understanding of the components that can shape the online learning climate and how it can be related to other psychological and behavioral variables (Kaufmann et al., 2015; Kaufmann and Vallade, 2022). This model proposes that there are first-order variables, such as teacher behavior, student characteristics, and structural-instructional aspects of the course, which influence second-order variables, such as student perceptions associated with self-efficacy, self-concept, or general beliefs about the learning process, and these in turn influence relevant variables in schools such as learning, critical thinking, and time use (Weber et al., 2011).

Teacher behaviors refer to the actions adopted to establish effective and affective interactions with their students, which, for Myers et al. (2018), requires two perspectives: rhetorical and relational. On the one hand, the instructor’s rhetorical perspective is focused on communicating the course content most clearly and understandably to contribute to learning achievements and academic grades, thus referring to the more objective aspects of the instructional process. On the other hand, the relational perspective aims to develop and maintain connections and relationships within the classroom over time, i.e., these objectives are associated with the effect of interpersonal connections that ultimately represent the subjective dimension in virtual learning contexts (Mottet et al., 2006; Frisby and Martin, 2010; Frisby et al., 2013, 2014; Frisby and Gaffney, 2015).

The student characteristics component refers to those personal attributes that differentiate students from each other (Weber et al., 2011). These differential characteristics predispose to shaping each student’s perceptions of peers and teachers and include intellectual, motivational, and emotional abilities (McCroskey et al., 2006). Therefore, these characteristics also contribute to shaping the assessment made of the relationships established with other course participants, which is particularly challenging in an online classroom environment in which interactions do not usually occur in the same geographical space and often do not occur synchronously so that the impressions generated among participants are conditioned by a scenario in which feedback does not necessarily happen immediately (Serhan, 2010).

Finally, course-specific structural issues refer to formal aspects related to the content and structure of the course, mainly related to clear rules and instructions to establish a working agreement between teachers and students (Weber et al., 2011). The transparency of these aspects promotes a perception of fair treatment in students, which has been favorably linked to academic outcomes and learning (Mottet et al., 2006). In online learning, instructions have a fundamental role as they are the basis for participants to understand the use of technological platforms and the operating guidelines in classes and the evaluation system (Kaufmann et al., 2015).

### Measurement of online classroom climate

The measurement of online classroom climate is mainly addressed using informatics, as it is a necessary resource for its implementation (Kaufmann et al., 2015; Bogolepova, 2021). Previous studies have provided insight into aspects related to online classroom climate; however, they present methodological limitations that impede the reporting of psychometric properties. For example, the Alqurashi (2019) and Cole et al. (2019) studies demonstrated the role of instructors and students in creating classroom climate environments, however, their sample size was small. In other cases, such as Swain et al. (2021), the participants belonged to computer science institutes, therefore, being related to a specific discipline it is not possible to generalize the results to other professions. Likewise, it is important to emphasize that for the measurement of variables of the educational context in virtual environments, it is not only enough to apply digital instruments, but it is also necessary to adapt them to the online environment, considering that many of these instruments have been developed to be applied in face-to-face classroom contexts (Hoi, 2022).

Therefore, evaluating the online learning climate from various perspectives and not only an informatic context is necessary. The educational context and the associated roles and interactions in this environment must also be evaluated. Previous literature, shows that few valid and reliable instruments measure classroom climate in virtual settings, one of them being the Online Learning Climate Scale (OLCS) by Kaufmann et al. (2015).

The OLCS is based on the premise that classroom climate comprises instructor behavior, student characteristics and behaviors, and course design/structure elements. Thus, this instrument explores how these dimensions articulate to establish the perception of classroom climate in online contexts (Kaufmann et al., 2015). The scale uses the IBM as its theoretical framework (Weber et al., 2011), and the initial bank of its items was created from the Classroom Communication Connectedness Inventory (Dwyer et al., 2004) and the Classroom Climate Scale (Gokcora’s, 1989).

This measurement was developed by Kaufmann et al. (2015), who, in a first study, generated a total of 47 items corresponding to the components of (1) instructor behaviors, (2) student characteristics and behaviors, and (3) specific structural aspects of the course; these items, in addition, were subjected to discussion in focus groups. The authors then conducted an exploratory factor analysis with a sample of 236 participants, from which they obtained the final version of the instrument composed of 15 items corresponding to four dimensions called Instructor Behavior (IB), Course Structure (CS), Course Clarity (CC), and Student Connectedness (SC). Subsequently, Kaufmann and Vallade (2022) conducted a confirmatory factor analysis of the scale, reaffirming the structure of four correlated factors. Although both studies showed excellent psychometric properties for the scale, they were developed with U.S. samples, so it would not be possible to guarantee *a priori* the cultural equivalence of the scale for its use in Latin America.

### The present study

Digitalization has impacted various areas of society, and education is not exempt from this since digital platforms have allowed the development of flexible teaching methodologies that do not require face-to-face attendance (Borrego et al., 2017). In this regard, although in European countries and the United States, distance education covers about 20% of enrollment in higher education, in Chile, the scenario is different because until 2018, the number of students enrolled in online programs was still incipient; however, due to the pandemic, institutions were forced to incorporate online learning tools considering the confinement policies in force at the time (Villarroel et al., 2021).

The Higher Education Information Service [Servicio de Información de Educación Superior (SIES), 2023] reported that there are currently 1,341,439 people enrolled in higher education institutions, of which 11.2% correspond to the distance mode, representing an increase of 24.9% compared to 2022. Therefore, one of the serious problems is attrition from some online programs (Lee et al., 2015; Bawa, 2016; Hsu et al., 2019). The associated difficulty is primarily with low engagement in online learning, as students generally feel isolated and detached from learning platforms (Lee et al., 2015; Hoi and Hang, 2021).

Previous studies have shown that classroom climate influences other variables, such as self-efficacy, self-esteem, and depressive symptoms in university students (Hong et al., 2021). Notably, in online learning, it has been evidenced that a greater connection with other students and a clear course structure are linked to lower feelings of loneliness (Kaufmann and Vallade, 2022). Considering this, it is relevant to determine the elements that conform to the online classroom climate, the factors that promote positive climates, and how this influences student learning (Ko and Rossen, 2011; Ni, 2018).

Currently, little is known about what constitutes classroom climate in online learning contexts, which presents an opportunity for its study in South America, especially in Chile, where there are no valid and reliable tools to measure the learning climate in virtual educational environments. In addition, previous studies have shown that behaviors and perceptions in classes are different depending on gender (Lee and Mccabe, 2021; Koul et al., 2023); therefore, it is necessary to guarantee the metric equivalence of the measure between men and women so that possible differences obtained are directly due to the levels of the variable and not to metric equivalence biases.

**Figure 1:**
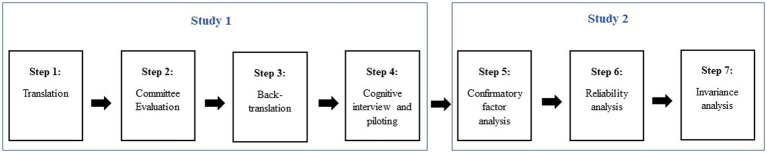
Flowchart of phases in the study.

Given this, the objective of this study was to culturally adapt the Online Learning Climate Scale (OLCS) and analyze its psychometric properties in Chilean university students. The present research was divided into two studies. The objective of the first study was to adapt the OLCS instrument linguistically and culturally in Chilean university students, while the second study had the objective of determining the internal structure, reliability indicators, and gender invariance of the questionnaire in a sample of Chilean university students. The steps followed in the two studies are specified in Figure 1.

## Study 1: linguistic adaptation and cultural relevance of the instrument

This study was conducted entirely online and following the guidelines on test adaptation that have been compiled by international organizations such as the International Test Commission^1^ and systematized in the scientific literature (Elosua et al., 2014; Muñiz et al., 2015). In addition, the research protocol was previously approved by the university’s Scientific Ethics Committee, in which voluntary participation was guaranteed through informed consent and data safeguarding under confidentiality and anonymity.

### Measure

The Online Learning Climate Scale (OLCS; Kaufmann et al., 2015) is a 15-item scale that measures online classroom climate through four dimensions, which are Instructor Behavior (IB), Course Structure (CS), Course Clarity (CC), and Student Connectedness (SC), with internal consistency indices ranging from 0.81 to 0.90. The response scale is a 7-point scale ranging from “strongly disagree” to “strongly agree.” Reliability in that study, estimated through Cronbach’s alpha, oscillated between 0.81 and 0.90.

### Participants

Expert committee: A multidisciplinary team comprising three professional translators from the university’s language coordination and research departments. Also, four researchers from the educational field: two English professors from the English Pedagogy degree program, a Doctor of Education with experience in evaluation instruments, and a PhD in Applied Linguistics. In addition, a psychologist with training in measurement.

Pilot test and cognitive interview: A sample of 13 participants between 18 and 21 years (61.5% female) was obtained through non-probability sampling. The inclusion criteria were to be a student enrolled and taking an undergraduate course at the university in virtual mode.

### Procedure

A robust linguistic and cultural adaptation procedure was performed through the application of the analytical-rational procedures for instrument adaptation (Muñiz et al., 2013, 2015; Elosua et al., 2014) to guarantee the adaptation and equivalence of the instrument.

Initially, the instrument’s authors were contacted by e-mail to obtain permission and guarantee the legality of the adapted version. Next, the translation process of the OLCS instrument was initiated through three independent translators from the original language, American English, to the target language, Spanish (forward translation). After that, the three versions were discussed by a team of four experts in education and a psychologist specialized in measurement. This committee of experts evaluated, first individually and then as a group, the translation of the instrument through verification criteria focused on the cultural relevance of the items and the equivalence of the items for the Chilean context (see Supplementary material 1). The instrument was considered culturally relevant and coherent for measuring the dimensions proposed in the theory (Elosua and López-jaúregui, 2007; Hambleton and Zenisky, 2011).

Following the agreement of this committee, the version underwent a back-translation process by two native speakers to complement the study concerning grammatical and semantic equivalence, cultural relevance, linguistic appropriateness, format, and instrument design (Muñiz et al., 2013, 2015; Elosua et al., 2014). These translators also approved the translation’s equivalence based on standardized criteria (see Supplementary material 2). For this step, inter-rater concordance indices were calculated through Aiken’s V, which is considered adequate if it is greater than 0.70.

Finally, a cognitive interview was conducted with a small sample of undergraduate university students (*n* = 13) to pilot the instrument and ensure it was correctly understood; this included instructions, content, and type of response (Elosua et al., 2014) (see Supplementary material 3). These participants evaluated the scale’s usability using a guideline with a total score from 0 to 20, with 14 points or more being interpreted as high usability.

### Results

#### Linguistic, cultural, and usability adaptation

The main grammatical agreements were related to the differences between English and Spanish regarding personal pronouns and discourse markers for gender and number. In English, the third-person singular pronouns “he” and “she” refer to people according to gender. However, Spanish uses gendered pronouns for all people, including the third person singular “él” (he) for masculine and “ella” (she) for feminine. This can generate challenges in cross-cultural research when using translated scales (Padrón et al., 2017; Pérez et al., 2019).

The central language adaptations were implemented after the cognitive interview regarding linguistic adequacy. In this aspect, the adjustments were made in the Student Connectedness dimension, and they respond mainly to cultural elements that reflect the students’ perception of greater closeness to their peers: “mis compañeros (as)” (my classmates) instead of referring to “los estudiantes” (the students) as was proposed in the translation. The inter-rater agreement indices for translation and back-translation were.83 and.88, respectively, and are therefore considered adequate.

Likewise, the instructions and the response scale that accompany each of the four dimensions of the instrument were translated and adapted grammatically and linguistically to be understood by the target population. Regarding usability, the report indicated that the OLCS presents high usability because the average of the evaluations was 18.2 on a 20-point scale. The results show that the experience of answering the scale and the design were comfortable and accessible. In addition, the statements are clear and easy to understand, which reduces the response time required to complete it, which averaged 12 min. The process ended with a pilot version of the original 15 items adapted to Spanish (see Table 1).

**Table 1:** Original and adapted versions of the OLCS.

Original instrument	Instrumento adaptado al español
Online Learning Climate Scale (OLCS)	Online Learning Climate Scale (OLCS)
*Based on my online class interactions with the instructor, I perceived my instructor:*	*Basándome en mis interacciones en clases en línea, percibí a mi profesor(a) como alguien:*
As understanding.	Comprensivo(a).
As respectful toward me.	Respetuoso(a) conmigo.
As supportive.	Un apoyo.
As responsive (e.g., provides feedback on assignments).	Receptivo (por ejemplo, proporciona retroalimentación sobre las tareas).
As engaged in the course.	Comprometido(a) con la asignatura.
As approachable (e.g., someone I would email or visit in virtual office hours).	Accesible (por ejemplo, alguien a quien contactaría por correo electrónico o visitaría en horario de atención virtual).
*Based on my experiences with and perceptions of this online course:*	*Basándome en mis experiencias y percepciones de esta asignatura en línea:*
The design of this course encouraged student interaction with students.	El diseño de esta asignatura incentivó la interacción entre estudiantes.
The technology used in this course fostered collaboration among students.	La tecnología utilizada en esta asignatura fomentó la colaboración entre estudiantes.
This online course provided ample opportunities for communication among students.	Esta asignatura en línea proporcionó muchas oportunidades de comunicación entre estudiantes.
*Based on my experiences with and perceptions of this online course:*	*Basándome en mis experiencias y percepciones de esta asignatura en línea:*
The organization of the course was clear.	La organización de la asignatura fue clara.
The instructions for use of technology were clear.	Las instrucciones del uso de tecnología fueron claras.
The instructions for assignments were clear.	Las instrucciones de los trabajos fueron claras.
*Based on my online class interactions with students in my class, I perceive:*	*Basándome en mis interacciones en clases en línea con mis compañeros(as), percibo que:*
Students as respectful of one another.	Mis compañeros(as) son respetuosos(as) entre sí.
Students as cooperative with one another.	Mis compañeros(as) cooperan entre sí.
Students as comfortable with one another.	Mis compañeros(as) se sienten cómodos(as) entre sí.

## Study 2: psychometric analysis of the instrument

The sample of Study 2 was obtained through non-probability sampling. Four hundred ninety-one undergraduate students of the Universidad de La Frontera were between 18 and 25 years old (54.1% female). The inclusion criteria were to be a student enrolled and taking an undergraduate course at the university in virtual mode. The adapted version of the instrument obtained in the first study was applied to this sample through the QuestionPro® platform.

### Analysis plan

Following the recommendations of Ferrando et al. (2022), a preliminary analysis of the data was performed to determine the existence of missing data or outliers, the latter being determined by a *p*-value below 0.05 in the Mahalanobis distance. Next, the univariate descriptive statistics were explored to ensure that the items provided variability and that none of the response categories had zero value in the frequency of responses; likewise, the values of skewness and kurtosis were inspected, whose values between 1 and −1 were suggestive of univariate normality, a scenario in which conventional estimators could be used (Muthent and Kaplan, 1992).

Next, multivariate normality was explored through Mardia’s statistics, considering that the assumption was fulfilled when the *p*-value of these was above 0.05. Confirmatory factor analysis (CFA) was then performed, based on the original structure of the instrument using the maximum likelihood (ML) estimator or its robust alternative (maximum likelihood robust, MLR) in case of non-compliance with normality; the choice of this alternative is due to the most recent recommendations of Li (2021) concerning the extraction of polytomous data.

The fit of the measurement model was evaluated using conventional indicators such as chi-square (*χ*^2^), comparative fit index (CFI), Tucker-Lewis index (TLI), root mean square error of approximation (RMSEA), and standardized root mean squared residual (SRMR). To consider a good model fit based on the sample size and the number of observable indicators, non-significant *χ*^2^ values, CFI and TLI ≥ 0.94, along with RMSEA and SRMR <0.07, were used as reference; in addition, factor loadings (λ) should be greater than 0.40 (Hair et al., 2019).

Next, the reliability coefficients for the total scale and its dimensions were estimated using a conventional estimator such as Cronbach’s alpha (*α*); in addition, McDonald’s omega (*ω*) was incorporated as it is a more accurate estimator in multidimensional structures (Trizano-Hermosilla and Alvarado, 2016). The coefficients are acceptable above 0.70 and good above 0.80 (Campo-Arias and Oviedo, 2008).

Finally, and complementarily, sex invariance analyses were performed; invariance analyses incorporate equality restrictions between groups to different model parameters (factor loadings, intercepts, and residuals). In this study, configural, metric (also known as weak), scalar (also known as strong), and residual (also known as strict) invariance models were tested (Kline, 2015). The criteria when comparing models were the change in CFI (ΔCFI) and RMSEA (ΔRMSEA) since the classical criterion of change in chi-square is sensitive to sample size; two models are considered to differ from each other and, therefore, the level of invariance is rejected when ΔCFI >0.010 (Cheung and Rensvold, 2002) and ΔRMSEA >0.015 (Chen, 2007).

Descriptive and univariate analyses were performed in the SPSS v26.0 statistical program, while multivariate estimations were performed using the *lavaan* package in RStudio.

### Results

Preliminary analyses showed no missing data or outliers in the participants’ responses, whose frequencies and percentages are presented in Table 2. On the other hand, univariate and multivariate analyses showed that the principle of normality was not met, so the models were run using the robust alternative of the maximum likelihood estimator (MLR).

**Table 2:** Univariate analysis of scale items.

Item	Response options							*M*	*SD*	Sk	k
	1	2	3	4	5	6	7				
1	28	26	34	50	51	84	218	5.43	1.88	−1.01	−0.19
	**5.7**	**5.3**	**6.9**	**10.2**	**10.4**	**17.1**	**44.4**				
2	10	12	21	28	44	70	306	6.09	1.49	−1.76	2.34
	**2**	**2.4**	**4.3**	**5.7**	**9**	**14.3**	**62.3**				
3	49	34	42	31	60	68	207	5.14	2.11	−0.79	−0.80
	**10**	**6.9**	**8.6**	**6.3**	**12.2**	**13.8**	**42.2**				
4	43	25	40	40	52	76	215	5.28	2.03	−0.92	−0.51
	**8.8**	**5.1**	**8.1**	**8.1**	**10.6**	**15.5**	**43.8**				
5	35	25	22	36	38	53	282	5.66	1.96	−1.27	0.24
	**7.1**	**5.1**	**4.5**	**7.3**	**7.7**	**10.8**	**57.4**				
6	38	26	32	44	47	59	245	5.43	2.00	−1.02	−0.31
	**7.7**	**5.3**	**6.5**	**9**	**9.6**	**12**	**49.9**				
7	55	23	45	69	69	78	152	4.87	2.03	−0.61	−0.85
	**11.2**	**4.7**	**9.2**	**14.1**	**14.1**	**15.9**	**31**				
8	55	23	45	69	69	78	152	4.81	2.02	−0.53	−0.94
	**11.2**	**4.7**	**9.2**	**14.1**	**14.1**	**15.9**	**31**				
9	63	36	40	69	73	77	133	4.66	2.08	−0.48	−1.06
	**12.8**	**7.3**	**8.1**	**14.1**	**14.9**	**15.7**	**27.1**				
10	50	35	39	30	48	61	228	5.21	2.14	−0.84	−0.79
	**10.2**	**7.1**	**7.9**	**6.1**	**9.8**	**12.4**	**46.4**				
11	32	29	35	56	47	79	213	5.33	1.94	−0.91	−0.43
	**6.5**	**5.9**	**7.1**	**11.4**	**9.6**	**16.1**	**43.4**				
12	34	34	31	42	60	71	219	5.34	1.97	−0.94	−0.42
	**6.9**	**6.9**	**6.3**	**8.6**	**12.2**	**14.5**	**44.6**				
13	3	10	5	25	64	129	255	6.15	1.19	−1.79	3.59
	**0.6**	**2**	**1**	**5.1**	**13**	**26.3**	**51.9**				
14	14	9	22	60	84	113	189	5.61	1.52	−1.15	0.87
	**2.9**	**1.8**	**4.5**	**12.2**	**17.1**	**23**	**38.5**				
15	11	7	19	64	103	129	158	5.57	1.42	−1.06	0.99
	**2,2**	**1,4**	**3,9**	**13**	**21**	**26.3**	**32.2**				

M, mean; SD, standard deviation; Sk, skewness; k, kurtosis; percentages in bold.

First, a model with four correlated factors (oblique) was estimated according to the original structure of the instrument; this model obtained an excellent fit to the data [*χ*^2^ (84) = 189.628; *p* < 0.001; CFI = 0.979; TLI = 0.973; RMSEA = 0.051 (IC90% 0.044–0.059); SRMR = 0.028] and the factor loadings can be considered adequate (see Table 3). Given that the correlations between the first three factors were high (*r* > 0.80), it was plausible to hypothesize the existence of a unidimensional structure; however, this model had a poor fit to the data [χ^2^ (90) = 1044.597; *p* < 0.001; CFI = 0.794; TLI = 0.759; RMSEA = 0.147 (IC90% 0.139–0.155); SRMR = 0.025] and the factor loadings of items 12, 13, and 14 were below the established cutoff point (see Table 3).

**Table 3:** Factor loadings for the different models estimated through the CFA.

Item number	Oblique factors				One-dimensional	Second order				
	F1	F2	F3	F4	F1	F1	F2	F3	F4	GF
1	0.885				0.861	0.886				
2	0.796				0.774	0.796				
3	0.946				0.924	0.946				
4	0.909				0.899	0.908				
5	0.889				0.886	0.888				
6	0.908				0.892	0.909				
7		0.927			0.841		0.928			
8		0.938			0.832		0.937			
9		0.910			0.788		0.910			
10			0.919		0.868			0.919		
11			0.926		0.882			0.927		
12			0.926		0.871			0.925		
13				0.731	0.320				0.735	
14				0.892	0.331				0.888	
15				0.905	0.394				0.907	
IB										0.950
CS										0.878
CC										0.945
SC										0.410

IB, Instructor Behavior; CS, Course Structure; CC, Course Clarity; SC, Student Connectedness.

Following the recommendations of Zhang et al. (2016) to avoid underestimation of unidimensional models due to possible multicollinearity, a second-order model was estimated in which the four factors that compose the scale do not correlate with each other but are explained by a second-order latent factor; this model obtained an excellent fit to the data [χ^2^ (86) = 211.226; *p* < 0.001; CFI = 0.975; TLI = 0.969; RMSEA = 0.055 (IC90% 0.048–0.062); SRMR = 0.038]; in terms of factor loadings, all items loaded significantly on their factors and these in turn on the second-order factor (see Table 3). Considering the fit of all the models, the one that best reflects the behavior of the data and is the most parsimonious is the four-factor oblique model; therefore, the remaining analyses were conducted with this model. In addition, excellent average variance extracted indicators were obtained for Instructor Behavior (AVE = 0.792), Course Structure (AVE = 0.856), Course Clarity (AVE = 0.853), and Student Connectedness (AVE = 0.716).

The estimated reliability coefficients were excellent for the dimensions of Instructor Behavior (*α* = 0.955; *ω* = 0.959), Course Structure (*α* = 0.948; *ω* = 0.948), and Course Clarity (*α* = 0.947; *ω* = 0.946), and good for Student Connectedness (*α* = 0.892; *ω* = 0.877). Likewise, the invariance analyses reached the residual (strict) level by gender. This means that the scale allows reliable comparisons between men and women and affirms that the differences are due to the data and not elements inherent to the measuring instrument (see Table 4).

**Table 4:** Goodness-of-fit indices for comparing measurement models in invariance analysis.

	CFI	TLI	RMSEA [IC 90%]	SRMR	Contrast	ΔCFI	ΔRMSEA	Decision
M1. Configural	0.974	0.968	0.059 [0.050–0.067]	0.032	-	-	-	Accepted
M2. Metric	0.972	0.967	0.059 [0.051–0.067]	0.045	M2vsM1	−0.002	0.000	Accepted
M3. Scalar	0.970	0.967	0.060 [0.052–0.068]	0.046	M3vsM2	−0.002	0.001	Accepted
M4. Residual	0.968	0.967	0.059 [0.051–0.066]	0.046	M4vsM3	−0.002	−0.001	Accepted

## Discussion

According to Kaufmann et al. (2015), the OLCS explores how instructor behaviors, student characteristics and behaviors, and course design/structure elements articulate to establish the perceived classroom climate in online contexts. Likewise, in the context of Higher Education in Latin America, research on the online learning climate is scarce, and in Chile, there are no valid and reliable tools to measure it. Therefore, the present research was divided into two studies: (a) to adapt the OLCS linguistically and culturally for Chilean university students and (b) to psychometrically validate the OLCS instrument.

The first study provided a consensus version of 15 items, and there was no need to eliminate any of them. In the same way, the instructions and the response scale accompanying each of the four dimensions of the instrument were translated grammatically and linguistically adapted for comprehension. Thus, the linguistic and cultural adaptation process yielded an instrument that maintains the four dimensions, number of items, and instructions of the one reported by Kaufmann et al. (2015), which shows the equivalence of the instruments at the semantic level.

A culturally sensitive adaptation of the measurement instruments guarantees the reduction of biases. It allows the comparability of results between studies that may be carried out in different countries since it is based on a robust process that is not limited to the direct translation of the questions of the original questionnaire but contextualizes them by recognizing the semantic particularities of the language and culture to which the target sample belongs ((Muñiz et al., 2013, 2015; Elosua et al., 2014;). Therefore, having an adapted version that shows excellent usability indicators is the basis for future studies that wish to include the measurement of the construct of their models, especially when considering that much of the literature in this area has been developed in English-speaking countries (Okoye et al., 2023), so that the evidence obtained in this background is not completely extrapolated to the Chilean reality; therefore, studies are needed to account for the phenomenon in this context, being one of the first requirements, to have valid and reliable instruments.

The second study sought to validate the instrument psychometrically. The results showed that the OLCS instrument is a four-factor scale, according to the original structure of the instrument, which presented a better fit to the data than the unidimensional model and an alternative second-order proposal. These four dimensions account for relevant aspects within the learning beliefs model and incorporate objective (e.g., classroom norms) and subjective (e.g., interactions perception) elements of didactic interaction that become central to the development of an adequate classroom climate, especially in those contexts where the dynamics ultimately differ due to the lack of a common physical environment (Weber et al., 2011; Kaufmann et al., 2015).

Likewise, the OLCS with its 15 items proved to be reliable, even improving the indicators obtained in previous psychometric analyses (Kaufmann et al., 2015; Kaufmann and Vallade, 2022), and the analyses reached the highest level of invariance (residual) when restricting parameters between men and women. Consequently, it can be stated that this is an accurate instrument whose items are consistent with each other and that it allows future research proposals to compare latent averages between men and women with the assurance that differences are directly due to the trait measured and not to methodological artifices inherent to the research instrument (Kline, 2015).

From this perspective, the OLCS scale makes it possible to gather information on what is happening in virtual classroom learning environments and thus enhance learning in the virtual environment (Laurillard, 2012). Likewise, it represents inputs for developing lines of research and the possibility of developing guidelines and improvement resources for online teaching programs (Thomas, 2012; Whittaker, 2015; Aristovnik et al., 2020). From this perspective, understanding the effects of online classroom climate on learning contributes to improving the teaching and learning process and well-being in the online classroom for both students and professors, especially in the current post-pandemic context. The OLCS scale as a valid and reliable measure has the potential to collaborate in higher education with the main problems that the literature has shown in online learning environments, i.e., dropout rates (Lee et al., 2015; Bawa, 2016; Hsu et al., 2019) and low engagement (Lee et al., 2015; Hoi and Hang, 2021), and contribute to the development and management of a positive online classroom climate (Ko and Rossen, 2011; Ni, 2018).

## Limitations and future research

Although the present study has strengths, there are also some weaknesses, the first of which was the non-probability sampling, which prevents the generalization of the data, especially because the sample studied corresponds to a university that exceptionally maintained online classes since the modality of study is entirely face-to-face; therefore, future studies should test the scale in samples of universities oriented to distance education. Likewise, other scales were not included to analyze the convergent validity of the instrument with other measures. Considering those mentioned above, future studies with the OLCS should test its concurrent validity with measures such as loneliness, subjective well-being, or academic dropout. Likewise, future lines of work could make longitudinal measurements to increase the measurements’ precision and evaluate the measure’s stability, i.e., whether the measure is consistent and stable over time. Longitudinal measurements would make it possible to detect changes in the measured variable. Finally, considering that virtual education increasingly allows people to connect in different parts of the world, it would be interesting to study the invariance of the test between countries.

## Conclusion

The above evidence from the validity analyses supports that the OLCS is a scale that works as theoretically expected, and it can be concluded that it is a valid and reliable instrument to measure the different dimensions of the online learning climate in the Chilean context.

## Data availability statement

The raw data supporting the conclusions of this article will be made available by the authors, without undue reservation.

## Ethics statement

The studies involving humans were approved by Comité Ético Científico de la Universidad de La Frontera ACTA N° 062_21. The studies were conducted in accordance with the local legislation and institutional requirements. The participants provided their written informed consent to participate in this study.

## Author contributions

MB-S: Conceptualization, Funding acquisition, Investigation, Project administration, Resources, Validation, Visualization, Writing – original draft, Writing – review & editing. RM: Data curation, Formal analysis, Methodology, Software, Writing – original draft. OT-M: Data curation, Formal analysis, Methodology, Software, Supervision, Validation, Writing – original draft. MM-C: Methodology, Validation, Writing – review & editing. LC: Investigation, Methodology, Resources, Writing – original draft.

## Footnotes

^1^http://www.intestcom.org
