# Supplementary material for: Cultural adaptation and psychometric properties of the online learning climate scale for Chilean university students
Source: Front Psychol. 2024 Feb 14;15:1280311. doi: 10.3389/fpsyg.2024.1280311 (PMC10899396; doi:10.3389/fpsyg.2024.1280311)
Supplement: Supplementary file 1 [file Table_1.DOCX]

Supplementary Material 1:

Quality control guideline for the translation-adaptation of the items (Hambleton y Zenisky, 2011)

| **GENERAL ASPECTS** | **Agree** | **Disagree** | **Observation** |
| --- | --- | --- | --- |
| Does the item have the same or very similar meaning in the two languages? |  |  |  |
| Does the type of language of the translated item have a difﬁculty and familiarity comparable to that of the original language? |  |  |  |
| Does the translation introduce changes in the text (omissions, substitutions, or additions) that might inﬂuence the difﬁculty of the item? |  |  |  |
| **ITEM FORMAT** | **Agree** | **Disagree** | **Observation** |
| Is the item format, including physical aspects, the same in both languages? |  |  |  |
| Are the length of the statement and of the response alternatives, if any, of similar length in both versions? |  |  |  |
| Are the item format and the task to be performed by the respondent of similar familiarity in the two versions? |  |  |  |
| **GRAMMAR AND WRITING** | **Agree** | **Disagree** | **Observation** |
| Are there any modifications to the grammatical structure of the item, such as sentence placement or word order, that might make the item more or less complex in one version than in another? |  |  |  |
| Are there any grammatical clues that might make the item easier or more difficult in the translated version? |  |  |  |
| Are there any grammatical structures in the original version of the item that have no equivalent in the translated version? |  |  |  |
| Are there any references to gender or other aspects that may give clues about the item in the translated version? |  |  |  |
| Are there any words in the item that have a univocal significance, but in the translated version may have more than one significance? |  |  |  |
| Are there changes in punctuation between the two versions that might make the item easier or more difficult in the translated version? |  |  |  |
| **CULTURE** | **Agree** | **Disagree** | **Observation** |
| Have the terms used in the item in the original language been appropriately adapted to the cultural context of the translated version? |  |  |  |
| Are there cultural differences that have a differential effect on the likelihood of a response being chosen in the original and the translated version? |  |  |  |
| Are the concepts involved in the item at the same level of abstraction in the two versions? |  |  |  |
| Is the concept or construct of the item equally familiar and does it have the same significance in the two versions? |  |  |  |
