# Supplementary material for: Cultural adaptation and psychometric properties of the online learning climate scale for Chilean university students
Source: Front Psychol. 2024 Feb 14;15:1280311. doi: 10.3389/fpsyg.2024.1280311 (PMC10899396; doi:10.3389/fpsyg.2024.1280311)
Supplement: Supplementary file 2 [file Table_2.DOCX]

Supplementary Material 2:

Quality control guideline for back translation (Elosua et al., 2014).

| **GRAMMATICAL EQUIVALENCE**  *Native grammatical structures that do not necessarily have equivalents in the target language.* | **NO** | **Yes** | | **Observation** |
| --- | --- | --- | --- | --- |
| Is there a change in the format (e.g., order of sentence elements, statement/alternative agreement, sentence length, etc.) that would result in a change in item difficulty in any of the dimensions? |  |  |  | |
| **SEMANTIC EQUIVALENCE.**  *Equivalence between the connotative meanings of the word in the original language and the voice given in the terminal language.* | **NO** | **Yes** | **Observation** | |
| Are there differences or mismatches between the connotative meanings of the original and target versions? |  |  |  | |
| **CULTURAL RELEVANCE.**  *It analyzes whether there are voices that cannot be directly translated from one language to another due to a lack of equivalent referents.* | **NO** | **Yes** | **Observation** | |
| Are there items with a high cultural load, which prevent them from being transferred directly from one language to another due to the lack of equivalent referents? |  |  |  | |
| **LINGUISTIC ADEQUACY** | **NO** | **Yes** | **Observation** | |
| Do the adapted items consider the characteristics of the target population (undergraduate students)? |  |  |  | |
| Do the adapted items consider the adaptation of the language to the profile of the evaluated (undergraduate students)? |  |  |  | |
| Do the adapted items present an appropriate readability of the text, such as length of sentences, number of words? |  |  |  | |
| **FORMAT AND DESIGN** | **NO** | **Yes** | **Observation** | |
| Is there any correspondence in the physical appearance of the items in the original and adapted versions? |  |  |  | |
