# Supplementary material for: Cultural adaptation and psychometric properties of the online learning climate scale for Chilean university students
Source: Front Psychol. 2024 Feb 14;15:1280311. doi: 10.3389/fpsyg.2024.1280311 (PMC10899396; doi:10.3389/fpsyg.2024.1280311)
Supplement: Supplementary file 3 [file Table_3.DOCX]

Supplementary Material 3:

Usability

| **Criteria** | **Attitudinal Component** | | |
| --- | --- | --- | --- |
|  | **AFFECTIVE** | **COGNITIVE** | **TOTAL** |
| **Experience in responding to OLCS** | Responding to the Online Learning Climate Scale was comfortable. (If they answer 3, 2 points. If they answer 2, 1 point. If they answer 1, 0 points).  I thought it was a waste of time. (If they answer 3, 0 points. If they answer 2, 1 point. If they answer 1, 2 points). Reverse item. | I thought the scale addressed an important issue. (If they answer 3, 2 points. If they answer 2, 1 point. If they answer 1, 0 points).  I understood all the items on the scale. (If they answer 3, 2 points. If they answer 2, 1 point. If they answer 1, 0 points).  It was interesting to answer this scale online. (If they answer 3, 2 points. If they answer 2, 1 point. If they answer 1, 0 points). | 10 points |
| **Design of the OLCS Spanish version** | The design of the scale was very comfortable. (If they answer 3, 2 points. If they answer 2, 1 point. If they answer 1, 0 points). | It was easy for me to access the scale. (If they answer 3, 2 points. If they answer 2, 1 point. If they answer 1, 0 points).  The format of the scale is clear and easy to use. (If they answer 3, 2 points. If they answer 2, 1 point. If they answer 1, 0 points).  It was complicated to select the response alternatives. (If they answer 3, 0 points. If they answer 2, 1 point. If they answer 1, 2 points). Reverse item.  The instructions to follow were adequate and precise. (If they answer 3, 2 points. If they answer 2, 1 point. If they answer 1, 0 points). | 10 points |
| **TOTAL** | 6 | 14 | 20 |

Own elaboration based on Padrón et al., (2017) and Pérez et al., (2019).

**If the participant obtains:**

| From 0 to 6 points | From 7 to 13 points | From 14 to 20 points |
| --- | --- | --- |
| It corresponds to the degree of “low usability” | It corresponds to the degree of “medium usability” | It corresponds to the degree of “high usability” |
